# Retrograde axonal transport of autophagic vesicles and dynein-dynactin protein interaction are attenuated during aging in the rat optic nerve *in vivo*

**DOI:** 10.4103/NRR.NRR-D-24-01326

**Published:** 2025-08-13

**Authors:** Xiaoyue Luo, Jiong Zhang, Johan Tolö, Sebastian Kügler, Uwe Michel, Mathias Bähr, Jan Christoph Koch

**Affiliations:** Department of Neurology, University Medical Center Göttingen, Göttingen, Germany

**Keywords:** aging, autophagic vesicles, autophagy, autophagy-lysosomal pathway, axonal transport, dynein, optic nerve, p150Glued, retrograde transport, two-photon microscopy

## Abstract

Aging is characterized by a decreased autophagic activity contributing to the intracellular deposition of damaged organelles and macromolecules. Autophagy is particularly challenging in neurons since autophagic vesicles are formed at the axonal tip and must be transported to the soma where final degradation occurs. Here, we examined if axonal transport of autophagic vesicles is altered during aging. We employed two-photon microscopy for *in vivo* imaging in the optic nerve of young and aged rats. In old animals (> 18 months old), retrograde autophagic vesicle transport was significantly reduced with regard to motility and velocity. While activation of autophagy was decreased, expression of key proteins of the autophagy-lysosomal pathway including p62 and procathepsin D and the number of autophagolysosomes was increased. Maturation of autophagic vesicles was shifted to more distal regions of the axon and axonal lysosomal clearing was impaired. In a pull-down assay, the protein binding between dynein and dynactin was decreased by half, which could explain the retrograde axonal transport effects. Taken together, retrograde axonal autophagic vesicle transport *in vivo* is diminished during aging accompanied by decreased autophagy activation, alterations of the lysosomal pathway, and a reduced dynein-dynactin binding.

## Graphical abstract

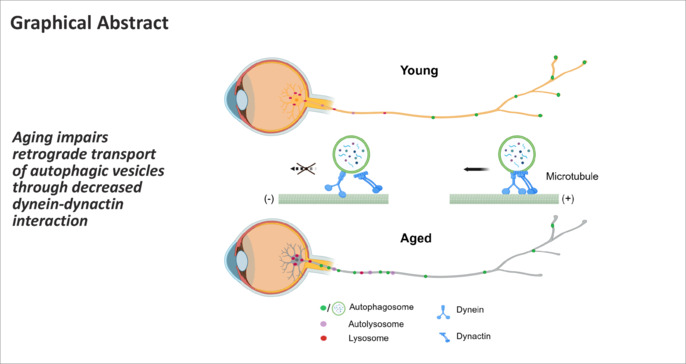

## Introduction

Aging is a physiological process that occurs in every biological organism, impairs vitality and ultimately leads to death. It is also the most important risk factor for most neurodegenerative diseases including Parkinson’s disease and Alzheimer’s disease (Azam et al., 2021) as well as for other pathologies such as cancer and cardiovascular disorders (López-Otín et al., 2023; Montégut et al., 2024). The molecular mechanisms underlying aging are heterogenous and comprise genomic instability, cellular senescence, mitochondrial dysfunction, and stem cell exhaustion in addition to others (López-Otín et al., 2023). One characteristic hallmark of aging is an impairment of autophagy that leads to a defective clearance of intracellular organelles and macromolecules which need to be degraded (Kitada and Koya, 2021; Palmer et al., 2025). Accordingly, autophagy holds promise as a therapeutic strategy for aging and various neurodegenerative diseases (Djajadikerta et al., 2020; Aman et al., 2021). However, it is not yet understood exactly which molecular pathways lead to the defected autophagy in aging and which contributing biological mechanisms play the major role in specific cell types.

Autophagy is a highly conserved metabolic pathway that mediates the transport of intracellular materials to lysosomes for degradation (Gómez-Virgilio et al., 2022). It plays a crucial role in maintaining cellular homeostasis by ensuring the quality control of longevity proteins and damaged organelles and by generating energy-rich compounds that can meet the bioenergetic demand of the cells during starving conditions (Wong et al., 2020; Cai and Ganesan, 2022). Macroautophagy (hereafter termed autophagy) starts with the formation of a phagophore engulfing the cytoplasmic material that is prone to degradation. The phagophore is elongated and finally closed around the material to form an autophagosome. Then the autophagosome fuses with a lysosome to form the autophagolysosome in which the luminal content is acidified and the engulfed material is finally degraded (Klionsky et al., 2021).

Neurons are highly polarized cells embedded in a complex differentiated cellular network. As postmitotic cells, they are particularly reliant on functional autophagy (Fleming et al., 2022).

A characteristic feature of neurons is their long processes, particularly the axon, which can extend over lengths exceeding 1 meter in humans. With regard to autophagy in axons, it has been shown earlier, that the majority of autophagosomes in neurons is continuously generated at the axon tips followed by transport back to the soma for further processing (Maday and Holzbaur, 2014). The transport of AVs containing aggregated proteins and dysfunctional organelles from distal axons toward the soma, where mature lysosomes are located, presents a distinctive challenge for neurons (Maday et al., 2012). The efficient transport of AVs relies on microtubule-based motor proteins, which are particularly important for substrate degradation (Nambiar and Manjithaya, 2024). Specifically, plus-end-directed motor proteins (kinesin-1) facilitate the transportation of autophagosomes to the cell periphery, while minus-end-directed motor proteins (dynein and dynactin) are responsible for moving autophagosomes toward the perinuclear region. During the retrograde movement, AVs undergo a gradual acidification and maturation process (Tempes et al., 2024). Finally, fusion with lysosomes is facilitated by both physical proximity and the deceleration of vesicle trafficking (Zaarur et al., 2014; Lőrincz and Juhász, 2020). Therefore, precise regulation of the transport process represents a crucial step in ultimately completing the autophagic metabolism.

However, although it is widely recognized that autophagic flux is reduced in an age-dependent manner in the nervous system (Wong et al., 2020), there is a lack of studies on axonal AV transport, primarily due to technical challenges in imaging axonal transport *in vivo*. In comparison to alternative methods, real-time observation of AV transport presents a more intuitive and accurate approach to identifying transport defects.

In this study, we have established an experimental setup enabling live imaging of axonal AV transport in the rat optic nerve *in vivo*. This methodology contributes valuable insights to our comprehensive understanding of aging processes. Additionally, we also provide new evidence on the impairment of the autophagy-lysosomal pathway, which is associated with transport defects observed in the optic nerve during aging.

## Methods

### Production of AAV.mScarlet-LC3

To visualize the movement of LC3-positive AVs *in vivo*, we produced adeno-associated viral vectors (AAVs) of the pseudotype 2/6 (AAV2/6) expressing the protein LC3 fused to the red fluorescent protein mScarlet.

The rat LC3 cDNA was amplified, fused with the cDNA for the red fluorescent protein mScarlet and cloned into an AAV-plasmid (pAAV). The resulting sequence for the LC3-mScarlet fusion protein was driven by a hybrid cytomegalovirus (CMV)/chicken beta-actin (CB) promoter and further enhanced by the woodchuck hepatitis virus post-transcriptional regulatory element (WPRE). The correct plasmid sequence was checked by PCR sequencing.

AAV were produced according to the method described by Zolotukhin et al (Zolotukhin et al., 1999). Briefly, Human embryonic kidney 293 (HEK293) (American Type Culture Collection, Manassas, VA, USA, Cat# CRL-1573, RRID: CVCL_0045) were transfected with calcium phosphate and a 0.5:0.5:1:1 molar ratio of pAAV-RC, pH21, pHELPER, and the LC3-mScarlet pAAV expression vector.

High-titer viral stocks were produced by FPLC with standard methods. Viral titers were determined by quantitative PCR.

### Animal experiments

Female Wistar rats weighing between 250–350 g, obtained from Charles River (Erkrath, Germany), were used for *in vivo* experiments. Only rats of one sex were used to exclude sex-related effects on the results and increase the homogeneity of the results since several previous studies demonstrated sex differences in particular on axonal de- and regeneration. Wistar rats used in this study were of specific-pathogen-free (SPF) grade. They were housed in individually ventilated cages (IVCs) with controlled temperature (22 ± 2°C), humidity (50%–60%), and a 12-hour light/dark cycle. Rats were group-housed (3–4 per cage). Anesthesia was induced with isoflurane and maintained using 10% ketamine (95 mg/kg) and 2% xylazine (7 mg/kg).

The study included both 3-month-old adult rats and rats over 18 months old. All animals were housed in a room with a 12/12 hour light and dark cycle and provided *ad libitum* access to standard laboratory food and water. Animal experiments were conducted with the approval of the local animal research authorities (LAVES, Oldenburg, Germany; approval number 17-2642, approved on December 9, 2017) and adhered to the legislation governing the management and care of laboratory animals in the state of Lower Saxony, Germany.

### Intravitreal injections of adeno-associated viral vectors

AAV.LC3-mScarlet was intravitreally injected into the left eye while the animals were under deep anesthesia. Anesthesia was induced with isoflurane (Zoetis, Leatherhead, Surrey, UK) via inhalation and maintained with an intraperitoneal injection of 10% ketamine (95 mg/kg; Bremer Pharma GmbH, Warburg, Germany) and 2% xylazine (7 mg/kg; Bayer HealthCare AG, Leverkusen, Germany). To achieve adequate optic nerve labeling with minimal toxicity, the virus titer was optimized. Subsequently, the most suitable concentration of 5.4 × 10^7^ TU per eye was applied using a Hamilton syringe 3 weeks prior to the optic nerve surgery.

### Optic nerve surgery

The exposure of the optic nerve was carefully carried out under a stereomicroscope as described previously (Koch et al., 2011). Briefly, under deep anesthesia, a midline incision was made in the skin of the head to expose the orbit. A part of the lacrimal gland was removed. A small electric driller was utilized to carefully shape the orbital bone to optimize the positioning of the two-photon objective (W Plan-Apochromat 20×; ZEISS, Jena, Germany). Subsequently, the superior palpebral and rectus muscles were detached from their respective insertion points, and the eyeball gently rotated ventrally. Finally, the optic nerve was exposed through a longitudinal dissection of the optic nerve sheath.

### *In vivo* live imaging of autophagic vesicle transport

After optic nerve exposure, the anesthetized rat was transferred to a custom-built two-photon microscope (LaVision BioTec TriM Scope II, LaVision BioTec GmbH, Bielefeld, Germany). Body temperature was maintained using a heating pad, and vital signs (heart rate, peripheral oxygen saturation, and body temperature) were closely monitored throughout the experiments. Anaesthesia was continuously sustained by intraperitoneal injection with minimal doses of 10% ketamine (95 mg/kg) and 2% xylazine (7 mg/kg).

An optical parametric oscillator (OPO, Chameleon, Coherent Inc., Santa Clara, CA, USA) tuned to 1100 nm for excitation was precisely focused onto the superficial layer of the optic nerve using a 20x water immersion Zeiss objective (W Plan-Apochromat 20x/1.0 DIC M27 75 mm; NA 1.0, Carl Zeiss AG, Jena, Germany). A Ti:Sapphire laser (Coherent Inc.), operating at a wavelength of 800 nm, served as the pump source. The imaging area was identified 1 mm away from the optic nerve head. To minimize artifacts resulting from animal respiration, a custom-designed respiration detector was incorporated into the experimental setup by placing an accelerometer on the thorax of the rat. This allowed for synchronization of the animal’s respiratory movements with image acquisition. A series of 200 time-lapsed images were obtained using ImSpector software (Abberior Instruments GmbH, Göttingen, Germany).

During imaging, a pre-warmed physiological solution was utilized to perform repeated rinsing of the orbit, ensuring the maintenance of a biological environment during live imaging. Additionally, the animal received subcutaneous injections of physiological solution at a rate of 1 mL per hour in the abdominal region to prevent dehydration.

### Analysis of *in vivo* live imaging

The raw live imaging time-lapse series of AV transport was opened in Fiji ImageJ (https://fiji.sc/), and the transport dynamics were subsequently quantified using a semi-automatically custom-built plugin. Automatic image registration was achieved through a combination of a custom-built macro and the TurboReg plugin (developed by Philippe Thévenaz (Biomedical Imaging Group, École Polytechnique Fédérale de Lausanne, Switzerland) (Thévenaz et al., 1998), followed by frame averaging and manual tracking of the AV’s movement trajectories to generate kymographs. Ultimately, the evaluation of the numbers of autophagosomes moving in different directions and those at rest, as well as the average velocity in each direction was done manually on the obtained kymographs. Only axons with moderate background fluorescence intensity and clearly distinguishable vesicles were included in the evaluation.

The kymographs were evaluated based on the following criteria: 1) AVs were tracked for a minimum of 70 and a maximum of 200 continuous frames (5–10 minutes); 2) for each video, 10 axons with identifiable AVs were evaluated; 3) LC3 vesicles moving less than a net 5 μm within a 3-minute time frame (net velocities ≤ 0.028 μm/s) were classified as stationary; 4) given the appearance of bulb-like structures in axons with age, trajectories with a diameter greater than 2 µm in the kymographs were not considered to be AVs and were excluded from the analysis, as they were more likely to represent degenerated bulbs or local axonal swelling (Vahsen et al., 2020); 5) each group included five independent animals.

### Immunohistochemistry

Animals were anesthetized as described above, followed by transcardial perfusion with phosphate-buffered saline (PBS, pH 7.4) and subsequently with 4% paraformaldehyde (PFA) diluted in PBS. The brain and optic nerve were carefully extracted and further fixed in 4% PFA overnight. Subsequently, the tissues were incubated in 30% sucrose at 4°C for at least 48 hours. Transverse sections of the superior colliculus (30 µm) and longitudinal sections of the optic nerve (16 µm) were prepared using a cryostat (CM3050, Leica, Wetzlar, Germany). Antigen retrieval was carried out in either cell conditioning solution (Ventana Medical Systems, Innovation Park Drive, Tucson, AZ, USA) (pH 8.5) or TBS (pH 9) (Sigma-Aldrich, St. Louis, MO, USA) at 50°C for 4 hours. To minimize background, 0.1% Sudan or 0.3 M Glycine (Sigma-Aldrich) was applied. Following incubation with blocking buffer containing 5% bovine serum albumin and 5% donkey serum for 1 hour at room temperature, sections were further incubated with the following primary antibodies: anti-cathepsin D (1:50, goat, Cat# STJ140018, St John’s Laboratory, London, UK), anti-GABA type A receptor-associated protein (GABARAP; 1:50, rabbit, Cat# ab109364, Abcam, Cambridge, UK), anti-LC3B (1:50, rabbit, Cat# L7543, Sigma-Aldrich), anti-phosphorylated ATG16L1 (p-ATG16L1; 1:50, rabbit, Cat# ab195242, Abcam), and anti-Unc-51-like kinase 1 (ULK1; 1:100, rabbit, Cat# A7481, Sigma-Aldrich). The corresponding secondary antibodies were then incubated with the sections at room temperature for 1 hour: Alexa Fluor 647-conjugated donkey anti-goat IgG (1:250, Cat# A21447, Thermo Scientific, Waltham, MA, USA), Alexa Fluor 488-conjugated donkey anti-rabbit IgG (1:500, Cat# A21206, Thermo Scientific, Waltham, MA, USA), and Cy3-conjugated donkey anti-rabbit IgG (1:500, Cat# 711-166-152, Dianova, Hamburg, Germany). Finally, the sections were mounted with Mowiol (Sigma-Aldrich). Imaging was performed using a Zeiss Axioplan microscope (Carl Zeiss AG) with a 63× oil immersion objective. The microscope was also equipped with an Apotome module, providing pseudo-confocal capability. All photographs were acquired at 0.24 μm intervals over a 2 μm distance using a motorized stage and merged by orthogonal projection to generate a single unified view of the tissue. For each group, data were collected from 3 rats, with 5 fields of view per rat, resulting in a total of 15 images per group.

### Image processing and quantification

For quantification, a machine learning-based image analysis tool, Ilastik, was employed to process the images through its pixel classification module, an easy-to-use interactive tool without requiring a high level of computational expertise (Berg et al., 2019). In brief, 3–4 images were selected from each group for training the software. Image features were defined by annotating the background and fluorescent particles. The images for evaluation were then batch processed using the defined parameters. The exported data were analyzed for particle counting with ImageJ and for colocalization with JACoP (Bolte and Cordelières, 2006).

### Western blotting

The optic nerves were dissected and homogenized using the previously described lysis buffer (Zhang et al., 2016). After centrifugation at 13,850 × *g* for 10 minutes, protein concentrations in the supernatant were determined by the bicinchoninic acid (BCA) assay (Cat# 23227, Thermo Scientific, Waltham, MA, USA). Subsequently, 20 μg of proteins were loaded onto either 4%–15% precast gradient gels (Bio-Rad Laboratories, Hercules, California, USA) or self-made gels and separated by sodium dodecyl-polyacrylamide gel electrophoresis (Bio-Rad Laboratories). The separated proteins were then transferred to polyvinylidene fluoride membranes (GE Healthcare). Following blocking with 5% bovine serum albumin at room temperature for 1 hour, the following primary antibodies were used for overnight incubation with membranes at 4°C: anti-LC3B (1:1000, rabbit, Cat# L7543, Sigma-Aldrich), anti-cathepsin D (1:300, mouse, Cat# SC-377124, Santa Cruz Biotechnology, Dallas, TX, USA), anti-SQSTM1/p62 (1:3000, rabbit, Cat# P0067, Sigma-Aldrich), anti-Dynein (1:500, mouse, Cat# MMS-400P, Covance, NC, USA), anti-p150Glued (1:500, rabbit, Cat# 69399, Cell Signaling Technology, Cambridge, UK), anti-Kif5 (1:1000, rabbit, Cat# 3500282, Sigma-Aldrich), anti-lysosomal associated membrane protein 1 (LAMP1; 1:200, mouse, Cat# SC-20011, Santa Cruz Biotechnology), anti-cation-dependent mannose-6-phosphate receptor (CD-MPR; 1:200, mouse, Cat# SC-365196, Santa Cruz Biotechnology), anti-actin (1:10000, rabbit, Cat# ab8227, Abcam), and anti-glyceraldehyde 3-phosphate dehydrogenase (GAPDH; 1:10,000, mouse, Cat# 5G4, Hytest Ltd., Turku, Finland). After washing in Tris-buffered saline/0.1% Tween-20 for 4 times, each for 10 minutes, membranes were further incubated for 1 hour at room temperature with the following corresponding horseradish peroxidase (HRP)-coupled secondary antibodies: anti-mouse HRP (1:3000, Cell Signaling Technology, Cat# 7076P2) and anti-rabbit HRP (1:3000, Cell Signaling Technology, Cat# 7074P2). Finally, the blots were developed with enhanced chemiluminescence (ECL) reagents (Amersham Biosciences, Cat# RPN2236, Cytiva, Amersham, UK), and the target band intensities were evaluated using Fiji ImageJ (Version 1.54), with the resulting outcomes standardized against GAPDH or actin as the loading control.

### Pull-down assay and immunoprecipitation analysis of the dynein and dynactin interaction

For immunoprecipitation, protein A agarose beads (Cat# 9863, Cell Signaling Technology) were used (Dillman and Pfister, 1994). All steps were performed at 4°C or on ice. Briefly, optic nerves were homogenized in the lysis buffer (25 mM Tris-base, 50 mM NaCl, 2 mM EDTA, and 0.5% Triton X-100, pH 8.1) containing both protease and phosphatase inhibitor cocktails (Roche, Basel, Switzerland). The lysates were centrifuged at 13,850 × *g* for 10 minutes. For each group, 200 μg of supernatants were incubated overnight at 4°C with either p150glued antibody (Cat# 69399, rabbit, Cell Signaling Technology) or mAb IgG isotype control antibody (rabbit, Cat# 3900, Cell Signaling Technology) under gentle rotation. Subsequently, 200 µL of agarose bead slurry was added to the antibody-supernatant mixture for 4 hours at 4°C. The beads were washed four times with lysis buffer and then separated from the supernatants through centrifugation at 2000 × *g*. Proteins were eluted with 2× Laemmli buffer mixed with dithiothreitol (DTT) and further processed for the dynein western blot as described above.

### Statistical analysis

No statistical methods were used to predetermine sample sizes; however, our sample sizes are similar to those reported in previous publications (Luo et al., 2024). Statistical analyses were performed using GraphPad Prism 8 software (GraphPad Software, San Diego, CA, USA, www.graphpad.com). Comparisons between two groups were performed using an unpaired *t*-test or Mann–Whitney *U* test. Error bars represent means ± standard error of the mean (SEM) calculated from three independent experiments. Significance was determined when *P* < 0.05.

## Results

### Retrograde axonal autophagic vesicle transport is decreased in aged animals *in vivo*

**Figure 1:**
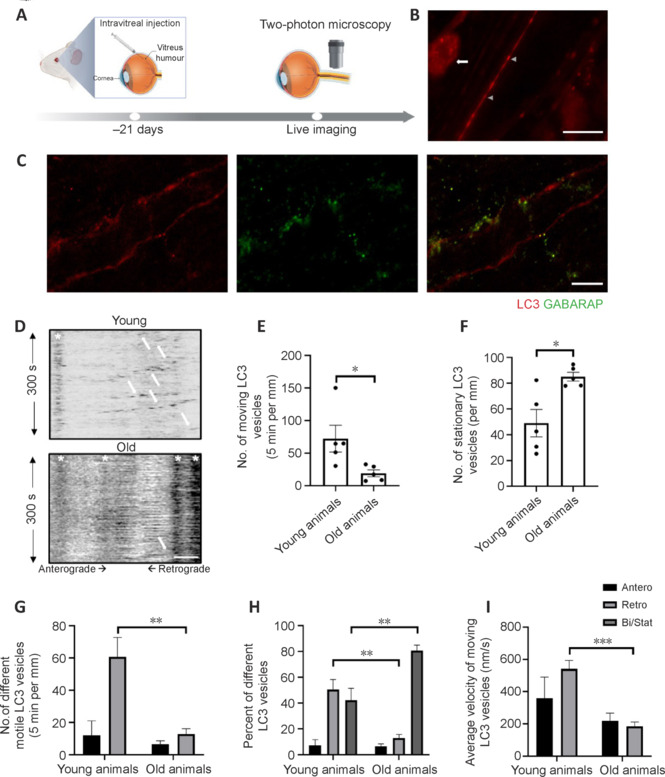
Axonal transport of autophagic vesicles in the optic nerve of young (3 months) and old (> 18 months) rats. (A) Experimental scheme of the *in vivo* live imaging procedure. AAV.mScarlet-LC3 was injected intravitreally into the left eye of rats. To achieve sufficient fluorescent labeling, a 3-week period was allowed for viral transduction. After surgical optic nerve exposure, the two-photon microscope was employed to acquire real-time imaging. (B) Flat mount section of the retina 3 weeks after AAV injection. The white arrow points to a retinal ganglion cell visualized by the LC3 virus. Gray arrowheads indicate exemplary individual autophagosomes in the axons. Scale bar: 10 µm. (C) Colocalization of LC3 virus-labeled autophagosomes (red) and GABARAP-positive puncta (green) in the optic nerve. Both LC3 and GABARAP belong to the ATG8 family and are involved in the process of autophagy. Immunofluorescence images demonstrate the co-localization of these two proteins, indicating that the LC3 virus effectively labels autophagosomes *in vivo*. Scale bar: 10 µm. (D) Exemplary kymographs of autophagic vesicles in the optic nerve of young (3 months) and old (over 18 months) rats. White arrows point at moving LC3 vesicles. Asterisks indicate stationary vesicles. Scale bar: 5 µm. (E–H) Quantification of the number and percent of motile and stationary LC3 vesicles in the optic nerve of rats at different ages. (I) Quantification of the average velocity of different moving LC3 vesicles in the rat optic nerve of given ages. In all quantifications, a minimum of 10 axons per time point per animal was evaluated. A minimum of 5 animals were included in each group. Error bars represent mean ± SEM. *P < 0.0, **P < 0.01, ***P < 0.001. In 1G and H, comparisons between the young and old groups for anterograde transport were performed using the Mann–Whitney *U* test. As for others, unpaired *t*-test was conducted. AAV: Adeno-associated virus; Antero: anterograde; Bi: bidirectional vesicles; GABARAP: GABA type A receptor-associated protein; Retro: retrograde; Stat: stationary vesicles;

To visualize axonal transport of AVs in young and old rats, we used our well-established *in vivo* live imaging set-up of the rat optic nerve (Knöferle et al., 2010; Koch et al., 2011). The optic nerve is an anatomically well-defined part of the central nervous system (CNS) that is easily accessible via transorbital surgical access. Adeno-associated viral vectors (AAV) of serotype 6 were constructed to express the protein LC3 fused with the red fluorescent protein mScarlet (AAV.LC3-mScarlet). Upon activation of autophagy, the cytosolic form of LC3 (LC3-I) is conjugated to phosphatidylethanolamine to form LC3-phosphatidylethanolamine conjugate (LC3-II), which is then recruited to autophagosomal membranes. Thus, in our model, mScarlet-labeled vesicular structures resemble LC3-positive AVs, which could be confirmed by a co-staining with the independent autophagosome marker GABARAP (**Figure 1C**; Varela et al., 2022), while a diffuse cytosolic background signal of mScarlet represents the cytosolic LC3-I isoform.

The AAV was injected intravitreally to transduce retinal ganglion cells (RGC) in young adult (age 3 months) and old (age 18–22 months) female Wistar rats (**Figure 1A** and **B**). Three weeks later, *in vivo* live imaging of the optic nerve was performed with a two-photon microscope. Videos of the labeled axons were acquired at a distance of 1 mm distal from the optic nerve head. The majority of the axons were labeled with the fluorophore and showed a diffuse background stain resembling cytosolic LC3-I. In several axons, vesicular structures could be identified and their movements could be tracked on kymographs (**Additional Video 1**). Since the AAV was injected intravitreally, all fluorophore-labeled structures in the optic nerve must be located within the RGC axons since this is the only anatomical connection between the retina and the optic nerve.

In young animals (3 months old), there was a relatively high mobility of AVs with more than 60% of all vesicles moving within an observation period of 5 minutes (**Figure 1D**). As expected, the vast majority was moving in the retrograde direction.

In aged animals (18–22 months old), there was a significant decrease in the number of moving AVs, from 72.3 ± 20.5 per mm in 5 minutes in young rats to 19.3 ± 5.2 per mm in 5 minutes in the aged rats (**Figure 1E**). Moreover, the number of stationary AVs significantly increased to 85.1 ± 3.4 per mm in the aged rats compared to 49.0 ± 10.6 per mm in the young animals (**Figure 1F**). This corresponds to a duplication of the percentage of stationary AVs during a five-minute observation period from 42% in the young animals to 82% in the aged animals (**Figure 1H**). Further analysis revealed that the reduction of moving AVs was caused by a specific decline of the retrograde transport which dropped from 60.8 ± 12.0 per mm in 5 minutes in the young rats to 12.8 ± 3.4 per mm in 5 minutes in the old animals (**Figure 1G**). The number of anterogradely transported AVs did not change significantly during aging.

The transport velocity of the AVs was also reduced in the aged animals, particularly in the retrograde direction. The average velocity of retrograde movement decreased from 540 ± 53 nm/s in the young group to 184 ± 28 nm/s in the old rats (*P* = 0.0003), while there was only a statistically non-significant trend for the velocity of anterograde movement in favor of the young animals (**Figure 1I**).

Taken together, the *in vivo* live imaging data demonstrate that in aged animals specifically, the retrograde axonal transport of AVs is significantly reduced with regard to vesicle motility and velocity in the rat optic nerve.

### Autophagosome formation at the axon tip is reduced in aged animals

**Figure 2:**
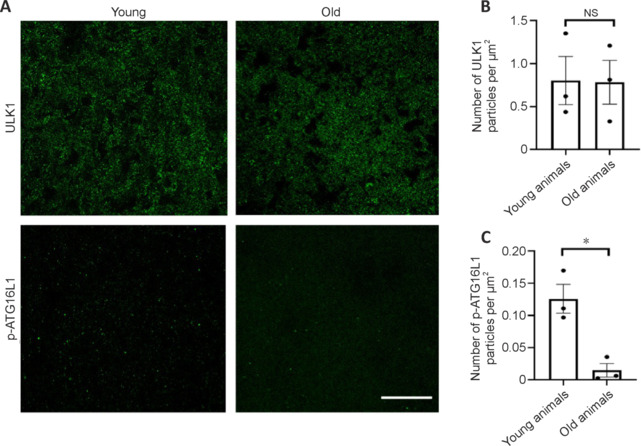
Autophagosome biogenesis in the SC of young and old rats. (A) Representative immunofluorescence staining of ULK1 and p-ATG16L1 in the rat SC at given ages. Scale bar: 50 µm. (B, C) Quantification of the number of ULK1 and p-ATG16L1 positive puncta per µm^2^ in the SC of young and old rats. Quantification was performed in the dorsal/superficial optic layer of the SC. Three independent experiments were included. Data were quantified from 3 rats in each group, comprising 15 images per group. Error bars represent mean ± SEM. *P < 0.05 (unpaired *t*-test). NS: Not significant; p-ATG16L1: phosphorylated ATG16L1; SC: superior colliculus; ULK1: Unc-51-like kinase 1.

We hypothesized that the reduced number of AVs being transported in the optic nerve in aged animals might be caused at least partly by a reduced formation of autophagosomes. Initiation of autophagy is mediated most commonly via the activation of the Atg1/ULK1 (Melia et al., 2020) complex, but alternatively also by ULK1-independent mechanisms (Gammoh et al., 2013). Both pathways lead to a p-ATG16L1 which is also involved in the conversion of LC3-I to LC3-II, with its levels being directly associated with the autophagy rate (Tian et al., 2020). In axons, autophagosomes are primarily generated at the axon tip (Maday et al., 2012). We thus performed fluorescent staining of both ULK1 and p-ATG16L1 in the optical layer of the superior colliculus (SC) which is the termination point of the optic nerve axons (**Figure 2**).

In the SC, we found no difference in the expression of ULK1 when comparing old to young animals (**Figure 2B**). The expression of p-ATG16L1 was significantly lower in aged rats compared to young rats (*P* = 0.01) (**Figure 2C**).

These data imply an impairment of autophagosome formation at the distal axon in the SC in aged animals via ULK1-independent pathways.

### Expression levels of key proteins of the autophagy-lysosomal pathways are increased in the optic nerve with age

Next, we analyzed expression levels of the key molecules that are involved in the autophagy-lysosomal pathway. Tissue protein lysates of the optic nerve of young and old rats were examined by Western Blotting.

**Figure 3:**
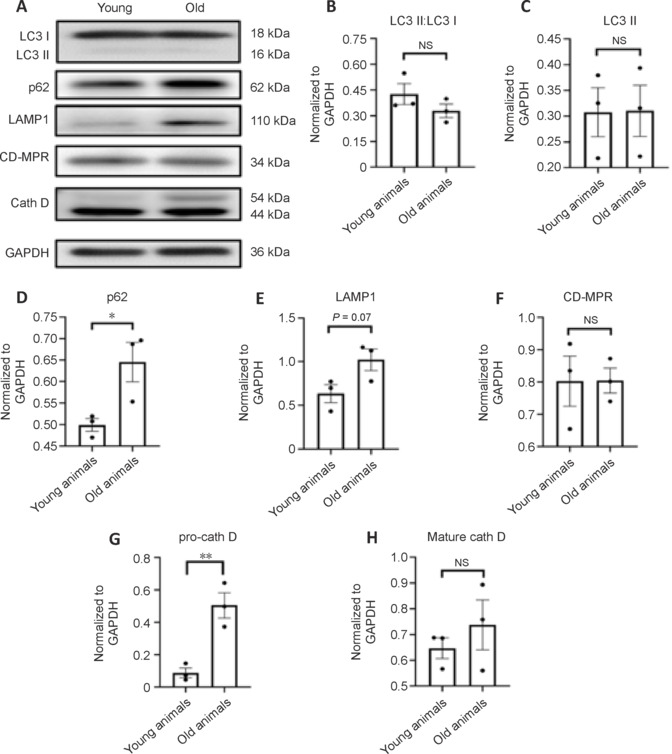
Alterations of autophagy-lysosomal pathways in the optic nerve of young and aged rats. (A) Representative immunoblots showing the expression levels of LC3, p62, LAMP1, CD-MPR, and cathepsin D in the optic nerve of young and old rats. (B–H) Quantifications of the band intensities of LC3 II:LC3 I, LC3 II, p62, LAMP1, CD-MPR, and cathepsin D normalized to GAPDH as loading control. Error bars represent mean ± SEM. At least three independent experiments were included. *P < 0.05 (unpaired *t*-test). cath D: Cathepsin D; CD-MPR: cation-dependent mannose-6-phosphate receptor; GAPDH: glyceraldehyde-3-phosphate dehydrogenase; LAMP1: lysosome associated membrane protein 1; NS: not significant.

LC3-I and -II levels as well as their ratio were not altered in old compared to young animals (**Figure 3A–C**). This finding might well reflect the impaired axonal transport that leads to a longer transit time and relative accumulation of autophagosomes in the optic nerve in old rats and could thus compensate for the decreased activation of autophagy in the SC of old animals, as shown above.

P62 (SQSTM1) is an autophagy-related protein that targets other proteins for selective autophagy (Kumar et al., 2022). In the optic nerve of aged rats, its expression levels were significantly increased (*P* = 0.04, **Figure 3D**). In the light of stable LC3-levels, this could indicate a defective autophagic flux, putatively driven by the impaired axonal transport of AVs. Alternatively, it could imply levels of proteins that are prone to degradation, or a combination of these two aspects.

Autophagosomes are progressively acidified on their way down the axon towards the soma and finally fuse with lysosomes for the ultimate degradation of their engulfed cargo (Kuijpers et al., 2021). A slowing of axonal autophagic vesicle transport could prepone acidification and lysosomal fusion to more distal areas of the axon. We thus examined the expression of several lysosomal proteins in the optic nerve.

LAMP1 is most commonly used as a marker for lysosomes (Cook et al., 2004; Saftig and Klumperman, 2009). Its expression in the optic nerve in the old versus young animals was statistically not significant (*P* = 0.07; **Figure 3E**).

The cation-dependent mannose-6-phosphate receptor, an oligomeric transmembrane protein responsible for the transport of lysosomal enzymes from the trans-Golgi network to endosomes/lysosomes (Olson et al., 2008), showed no significant difference between the two groups (**Figure 3F**).

Cathepsin D is an aspartyl protease that is translated as preprocathepsin in the endoplasmatic reticulum and then cleaved to procathepsin which is transported via the trans-Golgi network to the lysosomes. In the acidic lysosomes it is then activated to mature cathepsin (Yadati et al., 2020). In the aged animals, the expression levels of procathepsin (protein size: 54 kDa) were significantly increased 5-fold compared to young animals (*P* = 0.008). However, the expression of the mature cathepsin (protein size: 44 kDa) did not differ between young and old animals (**Figure 3G** and **H**). This finding could be best explained by an impaired cathepsin processing through lysosomes in the old animals, leading to an accumulation of the immature procathepsin.

Taken together, these results suggest an impaired intra-axonal degradation via the autophagy-lysosomal pathway in the aged animals as indicated by the increased p62 and (pre-) lysosomal marker levels.

### The number of autophagolysosomes is increased in aged animals

**Figure 4:**
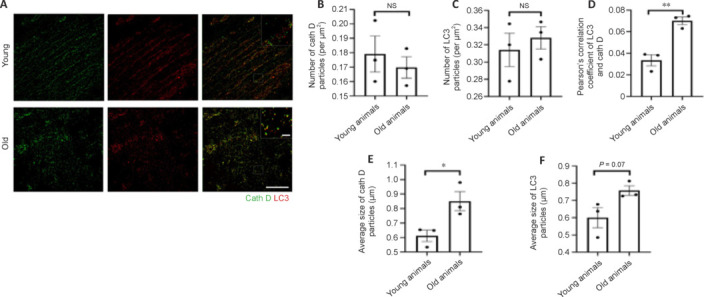
Immunohistochemical detection of autophagosomes, lysosomes, and autolysosomes in the middle optic nerve of young and old rats. (A) Representative immunofluorescence staining of cath D (green) and LC3 (red) in the rat optic nerve at different ages. Scale bar: 50 µm in the main image; 5 µm in the magnified boxes (upper right). (B, C) Quantification of the number of cath D and LC3 positive puncta per µm^2^ in the optic nerve of young and old rats. (D) Quantification of the colocalization of cath D and LC3 in the optic nerve at given ages (Pearson’s correlation). (E, F) Quantification of the average size of cath D and LC3 positive puncta in the optic nerve of young and old rats. Data were collected from 3 rats per group, with 5 fields of view per rat, resulting in a total of 15 images per group. Error bars represent mean ± SEM. Three independent experiments were included. *P < 0.05, **P < 0.01 (unpaired *t*-test). Cath D: cathepsin D; NS: not significant.

As the fusion of autophagosomes with lysosomes to become autophagolysosomes is a crucial step for the degradation of autophagy substrates (Zhao et al., 2021), we further investigated this process during aging. Autophagolysosomes are characterized by the co-existence of LC3 from the autophagosomes and active hydrolases from the lysosomes, with cathepsins being a major category. To identify autophagolysosomes in the optic nerve, we performed fluorescent staining of LC3 and cathepsin D and conducted colocalization analysis (**Figure 4A**).

As expected, we found almost twice as many LC3-positive autophagosomes as cathepsin D-positive lysosomes in the optic nerve, but for both there were no significant differences when comparing the different age groups (**Figure 4B** and **C**). Moreover, the proportion of autophagolysosomes as estimated by the Pearson’s correlation coefficient of LC3 and cathepsin D, was increased significantly in the old versus young rats, indicating an increased number of autophagolysosomes in aged animals (**Figure 4D**).

Interestingly, the size of cathepsin D-positive lysosomes was significantly larger in the optic nerve of aged rats (**Figure 4E**). Also, the size of LC3-positive AVs showed a directional increase of its diameter in the aged group which was, however, not significant (*P* = 0.07; **Figure 4F**). This is particularly interesting as it was shown before that the intracellular movement of lysosomes is inversely correlated with the organelle size (Bandyopadhyay et al., 2014).

In line with the lysosomal marker protein expression results above, these findings suggest that the impaired AV transport in the optic nerve of aged animals is accompanied by a shift of the fusion between autophagosomes and lysosomes to more distal areas of the axon and by alterations of lysosome size.

### The interaction between dynein and the dynactin complex is impaired in aging

Finally, we investigated molecular alterations that might underlie the prominent reduction in axonal AV transport in the aged animals.

Autophagosome motility relies on microtubule-based motor proteins. Kinesin-1 is essential for anterograde AV transport, whereas retrograde trafficking depends on dynein, a major minus-end motor protein (Wang et al., 2018). Dynein achieves efficient and robust retrograde transport by binding to p150Glued, the largest subunit of the dynactin complex, through the dynein intermediate chains (DIC). This interaction activates dynein, ensuring the long-distance retrograde transport of cargo (McKenney et al., 2014).

**Figure 5:**
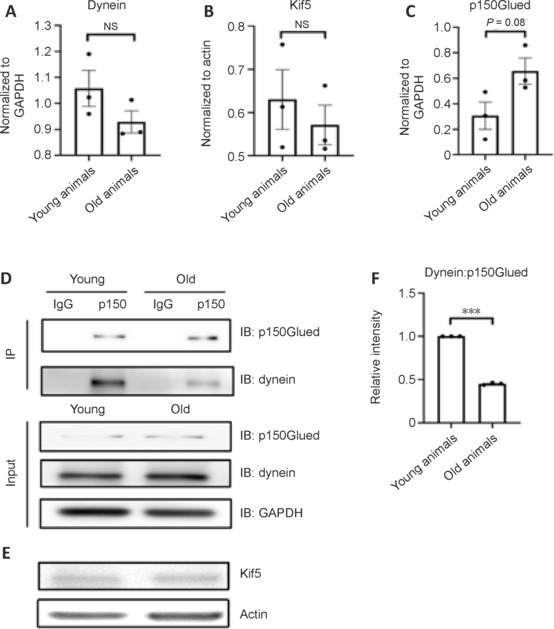
Alterations in motor proteins in the optic nerve of young and old rats. (A–C) Quantification of the band intensities of Dynein, Kif5, and p150Glued, and normalized to GAPDH or actin as loading control. (D) Representative immunoblots of immunoprecipitation illustrating reduced interaction between dynein and p150 glued in the optic nerve with aging. (E) Representative immunoblots showing the expression levels of kif5 in the optic nerve of young and old rats. (F) Quantifications of Western blot analysis following immunoprecipitation. The relative intensity of dynein was normalized to p150Glued. Error bars represent mean ± SEM calculated from three independent experiments. ***P < 0.001 (unpaired *t*-test). GAPDH: Glyceraldehyde 3-phosphate dehydrogenase; NS: not significant.

To assess age-related changes in motor proteins, we first compared the expression levels of these three key proteins in the optic nerve of young and old rats. Intriguingly, we observed no significant differences in the expression of dynein and KIF5, the most important member of the kinesin-1 family of proteins, with age (**Figure 5A**, **B**, and **E**). Also, expression levels of the largest dynactin subunit p150Glued only showed a directional increase in the aged animals without reaching significance (*P* = 0.08; **Figure 5C**).

Given the impaired retrograde axonal autophagic vesicle transport in aged animals, we hypothesized that the protein interaction between p150Glued and dynein might be affected. To test this, we performed a pull-down assay with the two proteins (**Figure 5D**). Here, we found that in aged animals the amount of dynein binding to dynactin p150Glued was only 50% of that in young animals (**Figure 5F**).

Thus, although protein expression of the dynactin subunit p150Glued is not changed in the aged animals, its protein interaction with dynein is strongly decreased. Since dynactin p150Glued is crucial for the dynein cargo interaction (Xiang and Qiu, 2020), the binding to microtubules and the activation of the retrograde motor protein complex, this reduced protein binding will most likely result in an impaired retrograde axonal transport in aged animals, as described above.

## Discussion

In this study, we observed a significant impairment of retrograde axonal AV transport during aging in the rat optic nerve *in vivo*, while anterograde AV transport remained stable. The protein interaction of dynein and dynactin, which is essential for retrograde transport, was reduced by half in aged animals, which could be an explanation for the impaired retrograde axonal transport of AVs. As expected, the amount of newly formed autophagosomes at the distal axon in the SC was reduced in aged animals. Molecular markers indicating a decreased degenerative clearance through the autophagosomal-lysosomal system were increased in the optic nerve with aging, including p62 and procathepsin. Moreover, there was an increased number of autophagolysosomes in the optic nerve in aged animals, indicating an earlier maturation of autophagosomes in the distal axon.

This is, to the best of our knowledge, the first published study analyzing axonal AV transport during aging in the CNS of a living mammal *in vivo*. There are, however, already several publications exploring effects of aging on axonal transport of different cargos *in vitro*, in explant models and in fixed tissue. Several early studies used radioactively labeled tracers to label axonal transport in general in animals and analyze the distribution post-mortem. Using 35S-methionine in the rat optic and sciatic nerve, a study found that the rate of fast and slow anterograde axonal transport decreased 40% between 6 and 24 months of age (McQuarrie et al., 1989). Similar results were achieved with 3H-leucine in the rat vagal and hypoglossal nerves and in ventral L5/6 spinal cord roots (Frolkis et al., 1997) and with radioactively labeled choline-phosphoglycerides in the rat sciatic nerve (Brunetti et al., 1987). A study using fluorescence live imaging of sciatic nerve and hippocampus fimbria explants from transgenic mice found that the anterograde and retrograde axonal transport of NMNAT2-vesicles and mitochondria was reduced in aged mice (6–12 months old) and even further in very old animals (24 months) compared to young adult animals (3 months) (Milde et al., 2015). Interestingly, the velocity of both transport compartments was not affected by aging in this model. In the proximal axons of RGCs imaged in the retina *in vivo*, axonal transport of mitochondria was altered with regards to duration, distance, and duty cycle but not with regards to net cargo flux and velocity in 23–25-month-old mice (Takihara et al., 2015). Moreover, *in vivo* live imaging of tetanus neurotoxin, which is transported retrogradely in endosomes, revealed no changes in axonal transport parameters in 13 months old mice compared with 1–8 months old mice in the sciatic nerve (Sleigh and Schiavo, 2016), but the animals examined in this study were relatively young. The only study so far looking at AV axonal transport in aging found an age-related decrease in the rate of AV formation at the axon tip and a decreased AV density and transport flux in the mid-axon in mice primary dorsal root ganglia neurons *in vitro* while AV transport in the distal and proximal axon were not severely altered with age (Tsong et al., 2023). Thus, our *in vivo* results are in line with most of these studies demonstrating a significant negative effect of aging on general axonal transport parameters. We confirm here, that this impairment also affects the transport of AVs, however, only in the retrograde direction. There was also a trend towards decreased anterograde transport with regard to net cargo flux and velocity in the aged animals, but without significance. Since the total amount of anterograde AV transport is much less than the retrograde direction, it is also possible that we did not detect an effect on anterograde transport due to statistical reasons.

Interestingly, we found also the velocity of retrograde axonal AV transport to be decreased significantly. Whereas the reduced AV flux could also be explained by decreased AV formation, the altered velocity indicates direct aging effects on axonal transport itself. Possible mechanisms include reduced binding to motor proteins, as evidenced in our study for dynein, and reduced energy supply by mitochondria or GAPDH (Chamberlain and Sheng, 2019). Since mitochondria are known to be affected by aging (Lima et al., 2022), it is very likely that this mechanism also contributes to our findings.

An age-related decline in autophagy activation and AV formation in neurons has been reported previously (Li et al., 2021; Tsong et al., 2023). Since the biogenesis of autophagosomes involves a series of steps, including initiation, nucleation, elongation, and closure (Melia et al., 2020), we examined two proteins associated with different early stages of autophagy formation in the SC. ULK1 mainly contributes to the initiation of autophagy, but ULK1-independent mechanisms can also mediate initiation (Gammoh et al., 2013). Moreover, p-ATG16L1 is more down-stream, crucial for autophagosomal membrane elongation and LC3 conjugation with phosphatidylethanolamine (Wang et al., 2018; Lystad et al., 2019). Interestingly, we found a significant reduction only of p-ATG16L1 but not ULK1. Our findings thus show that aging is associated with a reduced number of newly formed phagophores and autophagosomes at the distal axon which is putatively driven by ULK1-independent mechanisms. Alternatively, aging might not directly influence the initiation of autophagy, but rather impair the recruitment of factors necessary for phagophore elongation. This hypothesis is consistent with a previous study in mice dorsal root ganglia cells that demonstrated a normal AV initiation in aging, but subsequently a defective LC3-II recruitment (Stavoe et al., 2019). The specific molecular targets of aging in autophagy including putative changes in other ATG proteins need to be further analyzed in a future study.

To ultimately judge changes in autophagic flux, the application of drugs like bafilomycin to arrest AV-lysosome fusion would be necessary (du Toit et al., 2018). Unfortunately, this is not possible in our *in vivo* model since intravitreal or systemical application of such drugs would affect many different cell types leading to unpredictable side effects and an unspecific mode of action. Also, the time frame of such *in vivo* applications would be hard to determine. However, our findings that p62 levels are significantly increased together with stable LC3-II levels and decreased AV formation strongly suggest a decreased autophagic flux in aged animals which is in line with previous research in other models (Nieto-Torres and Hansen, 2021; Lim et al., 2024). A possible reason for the decreased autophagic flux is the impaired retrograde axonal transport which results in a reduced clearance of AVs, but additional, yet unknown factors that directly influence autophagic flux could also be involved.

The lysosomal pathways are also altered in aged *versus* young animals. Expression levels of the lysosomal marker LAMP1 and mature cathepsin D as well as the number of cathepsin D positive vesicles were not altered in the optic nerve of aged animals. There was, however, a strong significant increase in the expression levels of procathepsin D, which is the immature form of cathepsin. Assuming stable translation levels of cathepsin, this would mean that procathepsin is not processed as effectively anymore in aged animals, e.g. due to decreased clearance of AVs and lysosomes.

Our finding that only the retrograde transport of AVs is impaired while the anterograde transport remains unchanged suggests a specific effect of aging on the retrograde transport machinery. We observed here a decreased protein interaction between dynein and dynactin in aged animals which could well explain the defective retrograde transport. A reduced dynein-dynactin interaction with aging was found previously in whole-brain lysates from monkey brains (Kimura et al., 2007) and was shown to contribute to tau and amyloid precursor protein pathology (Kimura et al., 2016). Also, in aged oocytes the dynein-dynactin binding was reduced (D’Aurora et al., 2019). The mechanisms underlying the decreased dynein-dynactin binding with aging remain unclear. A possible explanation could be alterations in the phosphorylation state of dynein with aging which may contribute to the reduced binding efficiency of the dynein-dynactin complex (Gallisà-Suñé et al., 2023) as p150glued preferentially binds to the dephosphorylated dynein intermediate chains (Vaughan et al., 2001; Kimura et al., 2007).

Another contributing factor for reduced retrograde transport may be the age-related decreased density of microtubules and cytoskeletal rearrangements, which serve as the “tracks” for retrograde trafficking of cargo (Kim et al., 2022; Brill et al., 2023). Tubulin acetylation was shown to be upregulated in an age-dependent manner (Szyk et al., 2014) and excessive acetylation reduces the binding of motor proteins to microtubules (Geeraert et al., 2010; Kabir et al., 2022).

### Limitations

Despite the strengths of our *in vivo* imaging approach and the insights gained into autophagosome transport during aging, several limitations should be acknowledged. First, our study focused exclusively on the optic nerve, and the extent to which these findings generalize to other neuronal populations remains unclear. Second, although we observed correlations between aging, impaired retrograde transport, and reduced dynein-dynactin binding, causal relationships have not been directly tested and warrant further mechanistic investigation. Moreover, the functional consequences of impaired transport, such as its impact on neuronal integrity or visual function, were not examined. The necessary anesthesia of the animals might have altered the physiology of axonal transport, although no data support this so far. Finally, while two-photon imaging provides powerful real-time observation, it lacks the resolution to fully distinguish between autophagosome maturation stages or fusion events, underscoring the need for complementary ultrastructural analyses.

In summary, this is the first study of the effects of aging on axonal AV transport in the mammalian CNS *in vivo* revealing a specific impairment of the retrograde transport accompanied by a decreased dynein-dynactin interaction, a shift of AV maturation to more distal regions of the axon and a defective lysosomal clearing. Therefore, targeting the axonal transport machinery or enhancing autophagy-lysosomal function could be a potential therapeutic strategy to maintain neuronal homeostasis and delay neurodegeneration during aging.

## Additional file:

***Additional Video 1:*** *Representative two-photon live-imaging of the optic nerve in young and old rats. The somatic side is toward the left.*





## Data Availability

*All relevant data are within the paper and its Additional files*.
